# Three-Year Follow-Up of COVID-19 Cases in District of Constance, Germany. A Prospective, Controlled Cohort Study (FSC19-KN)

**DOI:** 10.3390/jcm14051439

**Published:** 2025-02-21

**Authors:** Ann-Kathrin Kohler, Stephan Richter, Michael Schmid, Heidi Zimmermann, Hannes Winterer, Steffen Schneider, Marc Kollum

**Affiliations:** 1 Hegau Bodensee Klinikum Singen, Gesundheitsverbund Landkreis Konstanz, Virchow Str. 10, 78224 Singen, Germany; kkohler@kohler-med.de (A.-K.K.); stephan.richter@glkn.de (S.R.); michael.schmid@glkn.de (M.S.); heidi.zimmermann@glkn.de (H.Z.); 2 Landratsamt Konstanz, Amt für Gesundheit und Versorgung—Gesundheitsamt, Schellestraße 15, 78315 Radolfzell, Germany; hwinterer@gmail.com; 3 Institut für Herzinfarktforschung, Bremserstr. 79, 67063 Ludwigshafen, Germany; schneider@stiftung-ihf.de

**Keywords:** COVID-19, post-COVID conditions, sequalae, virus variants

## Abstract

**Background and Objectives**: Long-term sequalae of viral diseases, especially after infections with SARS-CoV-2 (COVID-19), can induce multi-organ involvement, as around 65 million people worldwide report persistent symptoms that go far beyond the acute course. Studies indicate that early virus variants pose a higher risk of developing post-COVID-19 conditions. The primary aim of this study was to investigate the possible long-term effects based on the hospitalization rates and associated clinical events in patients infected with SARS-CoV-2 over an observational period of three years after the initial infection. Secondarily, an investigation of health-related quality of life and functional status was performed. **Methods and Materials**: The study presented was designed as a prospective, controlled cohort study to follow up on COVID-19 cases in the district of Konstanz, Germany (FSC19-KN). The positive group included subjects who had a primary infection with SARS-CoV-2 between March and December 2020. The control group included subjects who did not have a SARS-CoV-2 infection, as evidenced by a negative antibody test. As the primary endpoint, hospitalization rates and respective related admission diagnosis during the observational period of three years from January 2021 until July 2024 were analyzed. The health-related quality of life and functional outcomes were measured by the SF-36 questionnaire and Post-COVID-19 Functional Status (PCFS) as the secondary endpoint. **Results**: During the three years of observation after inclusion in the study, the hospitalization rate did not differ significantly between the two groups of initially infected and non-infected subjects (cumulative events, verum group 57 to control group 45, OR 1.24, CI 0.83; 1.85, *p* = 0.30). However, the health-related quality of life, measured by SF-36 sub scores of the SARS-CoV-2-positive subjects, achieved significantly lower results, except for the dimension ‘energy and fatigue’, in which subjects of the verum group still achieved significantly lower scores. **Conclusions**: Mild COVID-19 cases have no significant impact on hospitalization rates during an observational period of three years after initial infection. Yet, SARS-CoV-2-positive subjects reported a reduced health-related quality of life and functional outcomes. Ultimately, only the sub score quality ‘energy and fatigue’ still registered significant differences between both cohorts at the end of the three-year observational period.

## 1. Introduction

Coronavirus disease 2019 (COVID-19) caused by the severe acute respiratory syndrome coronavirus 2 (SARS-CoV-2) poses a challenge to health systems worldwide due to its ability to spread efficiently even in an increasing immunized population and its evolving resistance to currently available vaccines [1]. Adaptive mutations of the viral genome have the ability to alter its pathogenic potential and create new virus variants, where the replacement of even a single amino acid can drastically affects its ability to evade the immune system and complicates the development of effective vaccines [2,3]. In the past, Middle East Respiratory Syndrome (MERS-CoV) and Severe Acute Respiratory Syndrome (SARS-CoV) were zoonotic coronaviruses which, like the SARS-CoV-2 pandemic, caused severe respiratory diseases [4]. Particularly alarming are the long-term sequelae of respective viral infections, which remain a cause for concern despite available therapies and immunizations once infection has occurred [1]. Cells with ACE2 surface receptors are the primary targets [5] and are expressed throughout the human body, explaining the tropism of SARS-CoV-2 [3]. Clinically, many COVID-19 patients show mild to moderate symptoms without the need of hospitalization. However, some develop severe pneumonia, requiring mechanical ventilation with complications such as ARDS (Acute Respiratory Distress Syndrom), septic shock, and multi-organ failure, which are associated with high mortality rates [4,6]. Extrapulmonary manifestations of COVID-19 include acute kidney injury [3,7]. In a large multicenter cohort study of hospitalized patients, it was reported that 36.6% developed AKI (acute kidney injury), of which 14.3% required renal replacement therapy [8]. The effects of a SARS-CoV-2 infection on the central nervous system occur through the neuroinvasion of infected neurons via the olfactory nerve, via vascular endothelial cells, or through the migration of leukocytes across the blood–brain barrier [3,9]. It is assumed that direct viral cytotoxicity can cause damage to heart muscle cells. The release of pro-inflammatory cytokines such as IL-6 can lead to inflammation of the vessels, myocarditis, cardiomyopathy, and arrhythmias [3,10]. Patients with pre-existing endocrinological disorders, such as diabetes mellitus, have an increased risk of developing severe disease. Clinical manifestations such as abnormal blood glucose levels, euglycaemic ketosis, and diabetic ketoacidosis were reported in hospitalized patients [3,11]. In a multicenter meta-analysis by Elmunzer et al., gastrointestinal symptoms occurred in 53% of patients. The most commonly reported symptoms were diarrhea, nausea, vomiting, and abdominal pain [12]. Also, elevated liver function values are commonly found in 14% to 53% of patients with COVID-19 infection. Hepatic dysfunction is more common in patients with severe COVID-19 disease [13]. 

Many patients, regardless of disease severity, report persistent symptoms that go far beyond the acute course. More than 65 million people worldwide are affected by post-COVID conditions [14], which occur both after severe infection [4], but also after mild courses [15]. Findings suggested an increased risk for developing post-COVID conditions after infections caused by early virus variants than for those of the currently endemic Omicron variant [4].

## 2. Materials and Methods

### 2.1. Study Design

The prospective, controlled cohort study FSC-19-KN deals with the follow-up of patients infected with SARS-CoV-2 in the District of Constance (Baden-Wuerttemberg, Germany). It focused on the evaluation of consequential diseases, hospitalization rates, and the sequalae and its health-related quality of life of those affected in an ongoing research project lasting three years after their initial infection. This study was approved by the ethics committee of Albert Ludwigs University (Freiburg, Germany) and was registered on the German Clinical Trials Register (DRKS00022409, date of registration: 12 March 2020) and Clinicaltrials.gov (NCT04724434, date of registration: 13 January 2021).

**Figure 1:**
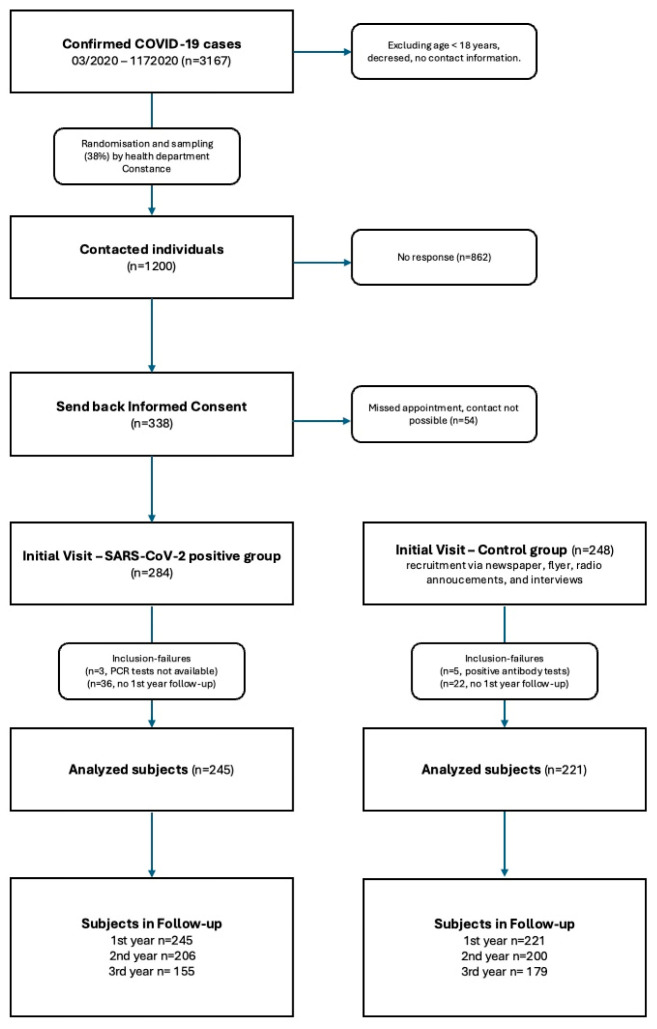
Recruitment of participants.

The recruitment of the SARS-CoV-2-positive group was performed in cooperation with the local health department (see Figure 1). A total of 1200 individuals were randomly sampled from all Polymerase Chain Reaction (PCR)-confirmed cases between March 2020 and December 2020 and contacted via mail. Specific causal variants were not documented. During initial visits between January and July 2021, 281 adults who fulfilled the eligibility criteria were enrolled at a mean of 203.5 days after infection. The common eligibility criteria were defined as follows: age ≥ 18 years, and the ability to read and sign the consent form and to grasp the nature of the study.

A total of 238 subjects exhibiting similar cardiovascular risk factors and negative SARS-CoV-2 antibody titers (Roche Elecsys Anti-SARS-CoV-2) were recruited as potential control partners via newspaper advertisements, flyers, radio announcements, and interviews [16].

Our analysis included 245 participants in the SARS-CoV-2 group and 221 participants in the control group. Participants who did not participate in the one-year follow-up were excluded. Since participants in the control group were infected with the SARS-CoV-2 virus during the follow-up, they were treated according to the intention-to-treat principle and remained in the control group accordingly.

The annual surveys were performed remotely (by mail, e-mail, phone, or via RED-Cap platform [17,18]).

### 2.2. Data Collection and Outcome Management

The study data were collected clinically by members of the study team of the Hegau-Bodensee academic teaching hospital (District of Constance, Germany). The physicians and acting principal investigators worked in the department of internal medicine. All data were collected and managed via RED-Cap [17,18], Research Electronic Data Capture, which is hosted on redcap.glkn.de. During the corresponding observation period, clinical events that have taken place were recorded to determine a possible correlation to the previous SARS-CoV-2 infection. Correlation assessment was conducted as follows: no correlation: no temporal connection, other diseases are to be assessed as the cause of the clinical event; possible correlation: existing temporal connection, other diseases may also be the cause of the clinical event; probable correlation: plausible temporal connection, other diseases cannot explain the occurrence of the clinical event; assessment not possible: insufficient information available.

In addition to clinical events, health-related quality of life was measured and analyzed using the Short Form Health Survey 36 (SF-36, see Supplementary Material).

### 2.3. Statistical Analysis

For the comparative presentation of cardiovascular risk factors, pre-existing medical conditions and sociodemographic data and COVID-19 disease-specific data descriptive statistics were used. Statistical analyses were performed with Stata/IC 16.0 for Mac (StataCorp, 4905 Lakeway Dr, College Station, TX 77845, USA). Difference in means and respective confidence intervals of 95% were calculated. Added odds ratio objectified the strength of association between events. Missing values were not included in the data analysis but were recorded accurately.

## 3. Results

### 3.1. Analysis of Study Population

The study population was composed of similar biometric data and demographics (Table 1).

Regarding the cardiovascular risk profile, both cohorts feature similar risk factors and comorbidities. Except bronchial asthma, which was the most common comorbidity in 22 subjects of the SARS-CoV-2 cohort with a percentage of 9% and was therefore significantly more common than in the control group (22 subjects or 9% vs. 8 subjects or 3.6%, OR 2.48, CI 1.08; 5.68, *p* = 0.03). The reported nicotine abuse in 91 subjects of the verum group vs. 86 in the control cohort stands out as the most frequent risk factor in the respective cohorts (Table 2).

Over the course of the observational period, the SARS-CoV-2 cohort showed lower reinfection rates compared to the control cohort, in which a significantly higher number of COVID-19 cases were recorded. At the first-year follow-up, 69 subjects of the control cohort (31.2%) reported being infected with SARS-CoV-2. After the second year, the number doubled to 121 participants (60.5%) (Table 3).

We observed a strong vaccination rate for the verum group in the first-year visit of 228 subjects (93.1%). However, this decreases significantly in terms of percentage in the booster vaccination. The first, second, and third booster vaccinations were carried out, respectively, by 16.3%, 0.4%, and 0% (Table 4).

### 3.2. Primary Endpoint

During the observation period, inpatient admissions were recorded with corresponding admission diagnoses (Table 5). Cumulatively, 57 (6.7%) clinical events occurred in the SARS-CoV-2-positive group, while 45 (5.5%) were reported in the control group. This shows a correspondingly even distribution pattern of clinical events (57 to 45, OR 1.24, CI 0.83; 1.85, *p* = 0.30). Table 5 covers the event classifications. Regarding the study focus, we compare the cardiological, pulmonological, and neurological clinical events in the respective follow-up from other disciplines (orthopedics, surgery, gastroenterology, ENT, urology, endocrinology, dermatology). The occurrence of cardiological, pulmonological, and neurological events was reported over the 3-year observational period in 21 events in the SARS-CoV-2-positive cohort while they occurred at 16 events in the control cohort, thus showing no statistical relevance (OR 1.27, CI 0.66; 2.46, *p* = 0.47). Furthermore, in the events of subspecialities orthopedics/surgery (24 vs. 17; OR 1.37, CI 0.73; 2.57, *p* = 0.32) and other subspecialities (12 vs. 12; OR 0.96, CI 0.43; 2.16, *p* = 0.93), no significant difference was noted.

### 3.3. Secondary Endpoint

In the initial visit, the evaluation of the health questionnaire short form (SF-36, Table 6) showed a statistically relevant correlation between having undergone SARS-CoV-2 infection and the reduced assessment of health-related quality of life in the verum group. Interestingly, however, as the study progressed, the data for both cohorts converged by the end of the two-year follow-up. Significant abnormalities were only found in the ‘Energy and Fatigue Score’, for which there was a clear progression of the difference in mean from 11.17 in the initial visit to 10.68 and 8.54 in the one-year and two-year follow-ups, respectively.

Initially, the functional state of health (PCFS-Scale) (Table 7) showed a significant difference (*p* < 0.0001), which, however, was already greatly reduced at the one-year follow-up visit (*p* = 0.012). At the end of the two-year follow-up, statistical significance was no longer detectable (*p* > 0.1).

## 4. Discussion

### 4.1. Cohorts

The matching procedure carried out after study inclusion was continued in the one-year follow-up and the corresponding data were compared with the entire subject pool. While the homogeneous study population of the matched cohorts were comparable in terms of age, gender, BMI, and relevant cardiovascular risk factors, the statistical comparison with the entire subject pool did not reveal any significant differences. As progressive subject dropouts are to be expected over the observation period of three years, the entire subject pool was analyzed in all three annual visits instead of matching cohorts. As a result, the significance of the resulting data increased due to a higher number of participants.

The occurrence of hypercholesterolemia and arterial hypertension were more common cardiovascular risk factors in the SARS-CoV-2-positive cohort compared to the control cohort. However, these differences were not statistically significant. In comparison, bronchial asthma in the SARS-CoV-2-positive subjects was statistically relevant. Otherwise, both cohorts exhibit a homogeneous distribution pattern regarding existing pre-existing conditions. Several studies have investigated the presence of bronchial asthma in connection with an increased risk of SARS-CoV-2 infection and a severe course of the disease. Yet, asthma has not been confirmed as an independent risk factor for either. Studies showed that the use of inhaled corticosteroids (ICSs) has been proven to be safe for asthma patients with COVID-19. It is also assumed that the use of ICSs could even offer protection against infection due to the reduced expression of transmembrane protease serine and ACE2 [19,20,21,22]. On the contrary, systemic corticosteroids must be considered as a risk factor for morbidity and mortality in asthma patients if they are used repetitively or chronically [20]. A meta-analysis in 2022 reviewed possible correlations of patients with AIRD (autoimmune rheumatic diseases) under treatment with DEMARD (disease modifying anti-rheumatic drugs) and susceptibility and clinical severity of COVID-19 due to the immunosuppressive effects DMARD therapy. They detected no significant differences between patients with or without AIRD concerning COVID-19 susceptibility, outcome severity, and morbidity. Furthermore, for steroid or DMARD therapy, no associated severe clinical outcomes were reported [23].

Studies showed 20–70% diagnosed patients were primarily asymptomatic on initial examination. After at least seven days of follow-up, most of them reported initial symptoms. Just under 20% remained asymptomatic throughout the course of the disease [24,25].

Our study population showed a low percentage hospitalization rate of the SARS-CoV-2-positive cohort and, compared to the data reported for the corresponding periods of the one-, two-, and three-year follow-up in Germany, an overly mild course of the disease.

High basic immunization rates are contrasted with a low percentage (re-)infection rate in the one-year follow-up. Drastically lower vaccination rates in single digits were recorded in the two-year follow-up. Yet, increased reinfection rates were documented in the two-year follow-up, especially in the control group. This may indicate a possible correlation [26].

The monitoring of the COVID-19 vaccination program of the Robert Koch Institute (RKI) also describes a declining vaccination rate in Germany at the time of the second booster vaccination, which remains at a low level even in high-risk groups over the age of 60. Even though, according to the results of the RKI’s COViK study, a booster vaccination significantly reduces the risk of hospitalization and a severe clinical course.

The post-COVID functional health status was statistically relevant at the initial study inclusion. Patients in the SARS-CoV-2-positive group reported significant functional restrictions and limitations in everyday life, while subjects in the control group reported fewer or no such restrictions. In the one-year follow-up, the results were only minimally significant; thus, the two-year follow-up, on the other hand, no longer recognized any statistical significance. This can be explained by the natural infections in the control group, which occurred during the observation period and adapted to the subjectively assessed functional health status of the verum group. Regarding the course of the study to date, which extends from study inclusion in January 2021 to the three-year follow-up in July 2024, a considerable reinfection rate, assumably due to a lower vaccination rate, shows an equalization of the cohorts in terms of health-related quality of life. In the one- and two-year follow-up, significant subject dropouts were recorded, with twice as many in the SARS-CoV-2-positive group than in the control cohort. This could be due to the reduced vitality, with comparatively greater fatigue in the verum group, which could explain the lack of motivation to continue the follow-up study. The increased vigilance and faster response rate of the control cohort in contrast to the SARS-CoV-2-positive group was also striking, which was noted in both the one-year and two-year controls. Infections with early variants, such as those in the verum group in the first and second infection waves during the pandemic in Germany, caused more harmful long-term consequences [17]. Since being included in the study, new infections or reinfections also occurred in the control cohort. However, these were affected by later variants. According to studies, this can be attributed to the severity of the course of the disease, which was milder; thus, those infected with the later Omicron variant had fewer long-term sequalae [27].

In order to keep the response rates stable, the survey of the secondary endpoint, health-related quality of life, was omitted in the three-year visit.

### 4.2. Clinical Events

During the observation period of three years, the evaluation of clinical events with the necessity for hospitalization in the SARS-CoV-2-positive group and classified with a possible relation to a previous infection showed no significant relevance compared to the control group. Also, the occurrence of admissions in the sub specialty of cardiology did not produce relevant differences. Studies have shown that long-term cardiorespiratory symptoms tend to occur less frequently after an Omicron infection compared to infection with previous variants, while neurological symptoms remain prevalent [4,28].

The primary endpoint, with the question of more frequent occurrence of clinical events in the verum cohort, could therefore not be proven to be statistically relevant in the present observation period.

### 4.3. Health-Related Quality of Life

Post-viral fatigue has often been described in patients recovering from moderate and severe cases; Kashif et al. recognized similar sequelae even in mild forms of the disease [29]. Our study determined similar results, where, initially, all data described the subjective overall reduction in health-related quality of life, while maintaining physical performance. While all dimensions of the SF-36 showed statistically relevant differences between the cohorts in the initial visit, they were only partially significant in the one-year follow-up and no longer significant in the two-year follow-up. The only dimension, measuring the subjective assessment of the subjects ‘energy and fatigue’, was statistically relevant over the whole course of the study. A two-year follow-up study of patients with post-COVID-19 condition was conducted in Sweden, which also found persisting symptoms after the observational period, showing a need for rehabilitation following infections with SARS-CoV-2 [30]. Here, the perception and self-assessment of the verum group continued to be significantly worse. The timing of the infections may explain why a large part of the health-related quality of life leveled off over the course of the study, consequently also due to (re-)infections of the test subjects.

Although many studies have described the general consequences and long-term effects of an infection with COVID-19, there has hardly been any research to date that addresses the connection between genetic changes or mutations of the virus and the development of long COVID [14]. According to Crook et al., earlier variants can cause more harmful long-term consequences [31]. The relationship between risk factors, comorbidities, and vaccination status with an increased risk of long COVID has rarely been investigated [4]. Wynberg et al. found no evidence that basic immunization improves the symptoms of existing long COVID [32]. A retrospective study published in February 2023 found that patients infected with the Omicron variant were less likely to develop long-term consequences of COVID-19 than patients infected with earlier variants [4]. This is consistent with the results of our study, in which subjects after study inclusion and infection of the wild-type SARS-CoV-2 and the alpha variant in 2021 showed a statistically relevant difference between the two cohorts in terms of health-related quality of life at the initial study entry visit and the follow-up visit of the first year. Subsequently, for all dimensions except for ‘the energy and fatigue score’ adapted during the two-year follow-up visit, which included the infections of the delta and omicron variants, no significant difference was recorded. Jong Mi Park et al., who studied neurological disorders during the pandemic, also reported a higher prevalence of stroke in later variants versus the original strain of SARS-CoV-2 [33].

A recent study from Spain found an increased risk for ICU admission and COVID-19-related mortality during the alpha wave, compared to the time period when the delta variant predominated [31]. In 2022, researchers at Vanderbilt University Medical Center in Nashville, TN, USA, used whole genome sequencing to suggest that the severity of COVID-19 disease progression was significantly milder in the Omicron variant compared to the identified alpha and delta variants [29], which explains the lower incidence of long COVID symptoms [34]. Antonelli et al. examined variants in different infection periods and found fewer long COVID cases after infection with Omicron compared to infections with earlier variants [35].

## 5. Conclusions

Sequalae that affect organ systems after viral infections are also described in the case of COVID-19. The present controlled follow-up study investigates a potential increase in incidence of SARS-CoV-2-related hospitalizations, as well as the effects on health-related quality of life of residents of the district of Constance, over an observation period of three years. This study refers to data collected during the follow-up visit of the first, second, and third year after acute infection, including an assessment of clinical events and questionnaires on health-related quality of life.

The study cohort was predominantly characterized by mild disease progression, which had not been selected accordingly at the time of study inclusion. Inpatient admissions were low in both cohorts and not statistically relevant. However, some clinical events in the SARS-CoV-2-positive cohort were assessed with a ‘possible’ or ‘probable’ relation to previous infection. Subjects in the control group also experienced clinical events in the further study course, which were possibly related to natural occurring infections during the observation period. Initially, the subjects in the SARS-CoV-2 group had significantly lower scores of health-related quality of life, which, due to reinfections and infections in the control cohort, became less significant as the study progressed. The dimension of ‘vitality’ (energy and fatigue) remains an exception, which may be explained due to the different causal virus variants that triggered the respective infection and accordingly entail more harmful or milder disease progression and sequalae. However, the consequences of viral infectious diseases are fundamentally attributable to multifactorial pathomechanisms.

In summary, collective results show that a SARS-CoV-2 infection with a mild course does not significantly affect the hospitalization rate three years after primary infection. However, there were significant reductions in health-related quality of life in early infections of the pandemic during the observation period, which are attributable to infections caused by the wild type and the alpha variant. It can be assumed that later infections, which include those of the delta and omicron variants, result in fewer long COVID cases due to their milder disease course and have fewer effects on the secondary endpoint, which were no longer statistically relevant in the comparison of the cohorts in the third year follow-up.

## 6. Limitations

There are several limitations that must be considered when interpreting the findings of this study. First, the study’s monocentric design, confined to the Constance district, may limit the broader applicability of the results, particularly due to potential regional differences in healthcare delivery and demographic factors. Second, the exclusion of quality of life measurements from the third year of follow-up, while necessary to ensure high participation rates, creates an incomplete picture of long-term outcomes, especially given that quality of life was a key outcome of the study. Additionally, the potential impact of multiple testing on statistical significance, particularly in subgroup analyses of different clinical events, may have increased the risk of false positives.

The approach to handling missing data makes it difficult to assess the potential biases introduced by differential loss to follow-up between groups. Furthermore, while the attribution of differences in outcomes could be linked to viral variants, this inference is based more on temporal associations than on direct identification of the variants, introducing a degree of uncertainty and confounding factors such as changes in treatment protocols or preventive measures over time, which may influence the results.

Finally, the analysis of vaccination effects is limited by incomplete data on booster doses and fails to consider the timing of vaccinations in relation to infection, which may influence the observed results.

## Figures and Tables

**Table 1:** Study population.

	**SARS-CoV-2 Cohort**	**Control Cohort**	**Missing Values**
	initial visit * n = 245	initial visit * n = 221	SARS-CoV-2/Control
	1st year ^ƒ^ n = 245	1st year ^ƒ^ n = 221	
	2nd year ^‡^ n = 206	2nd year ^‡^ n = 200	
	3rd year ^∆^ n = 155	3rd year ^∆^ n = 179	
**Age—Years**	46.6 ± 15.9 [44.6; 48.6] *	48.8 ± 14.2 [46.9; 50.7] *	-
	46.7 ± 15.9 [44.7; 48.7] ^ƒ^	48.8 ± 14.2 [46.9; 50.7] ^ƒ^	
	46.7 ± 15.7 [44.5; 48.8] ^‡^	49.6 ± 13.9 [47.6; 51.5] ^‡^	
	46.4 ± 15.7 [43.9; 48.9] ^∆^	49.9 ± 13.9 [47.8; 51.9] ^∆^	
18–39—No. (%)	87 (35.5) *	56 (25.6) *	-
	86 (36.2) ^ƒ^	56 (25.4) ^ƒ^	
	72 (35.0) ^‡^	47 (25.5) ^‡^	
	56 (36.2) ^∆^	40 (22.4) ^∆^	
40–59—No. (%)	107 (43.7) *	113 (51.1) *	-
	107 (43.9) ^ƒ^	113 (51.1) ^ƒ^	
	92 (44.7) ^‡^	104 (52.0) ^‡^	
	68 (43.9) ^∆^	95 (53.1) ^∆^	
60–79—No. (%)	49 (20.0) *	51 (23.1) *	-
	49 (20.1) ^ƒ^	51 (23.1) ^ƒ^	
	42 (20.4) ^‡^	48 (24.0) ^‡^	
	31 (20.1) ^∆^	43 (24.1) ^∆^	
>80—No. (%)	2 (0.8) *	1 (0.5) *	-
	2 (0.8) ^ƒ^	1 (0.5) ^ƒ^	
	0 (0.0) ^‡^	1 (0.5) ^‡^	
	0 (0.0) ^∆^	1 (0.6) ^∆^	
**Gender**			
Male—No. (%)	109 (44.5) *	94 (42.5) *	-
	108 (44.3) ^ƒ^	94 (42.5) ^ƒ^	
	90 (43.7) ^‡^	82 (41.0) ^‡^	
	70 (45.2) ^∆^	73 (40.8) ^∆^	
Female—No. (%)	136 (55.5) *	127 (57.5) *	-
	136 (55.5) ^ƒ^	127 (57.5) ^ƒ^	
	116 (56.3) ^‡^	118 (59.0) ^‡^	
	85 (54.8) ^∆^	106 (59.2) ^∆^	
**BMI—kg/m^2^**	25.3 ± 4.3 *	24.9 ± 4.4 *	-
	25.3 ± 4.3 ^ƒ^	24.9 ± 4.4 ^ƒ^	
	25.3 ± 4.4 ^‡^	24.9 ± 4.4 ^‡^	
	25.5 ± 4.7 ^∆^	24.9 ± 4.4 ^∆^	

Results are given as absolute value with (percentage)/mean ± standard deviation. (*) initial visit, (*^f^*) 1st year visit, (^‡^) 2nd year visit, (^Δ^) 3rd year visit.

**Table 2:** Cardiovascular risk factors and comorbidities.

	**SARS-CoV-2 Cohort**	**Control Cohort**	**Missing Values** SARS-CoV-2/Control
	initial visit * n = 245	initial visit * n = 221	initial visit *
	1st year ^ƒ^ n = 245	1st year ^ƒ^ n = 221	1st year ^ƒ^
	2nd year ^‡^ n = 206	2nd year ^‡^ n = 200	2nd year ^‡^
	3rd year ^∆^ n = 155	3rd year ^∆^ n = 179	3rd year ^∆^
Cardiovascular risk factors			
Diabetes mellitus —No. (%)	5 (2.0) *	5 (2.3) *	0/0 *
	5 (2.0) ^ƒ^	5 (2.3) ^ƒ^	0/0 ^ƒ^
	4 (1.9) ^‡^	5 (2.5) ^‡^	0/0 ^‡^
	2 (1.3) ^∆^	4 (2.2) ^∆^	0/0 ^∆^
Arterial hypertension —No. (%)	46 (18.8) *	32 (14.5) *	0/0 *
	46 (18.8) ^ƒ^	32 (14.5) ^ƒ^	0/0 ^ƒ^
	41 (19.9) ^‡^	30 (15.0) ^‡^	0/0 ^‡^
	32 (20.7) ^∆^	29 (16.2) ^∆^	0/0 ^∆^
Hypercholesterinemia —No. (%)	32 (13.1) *	21 (9.5) *	5/0 *
	32 (13.1) ^ƒ^	21 (9.5) ^ƒ^	5/0 ^ƒ^
	30 (14.6) ^‡^	20 (10.5) ^‡^	3/0 ^‡^
	25 (16.1) ^∆^	18 (10.1) ^∆^	3/0 ^∆^
Smoking —No. (%)	91 (37.1) *	86 (38.1) *	0/0 *
	91 (37.1) ^ƒ^	86 (38.1) ^ƒ^	0/0 ^ƒ^
	70 (34.0) ^‡^	79 (39.5) ^‡^	0/0 ^‡^
	56 (36.1) ^∆^	68 (38.0) ^∆^	0/0 ^∆^
Family history of coronary artery disease —No. (%)	41 (16.7) *	39 (17.7) *	5/3 *
	41 (16.7) ^ƒ^	39 (17.7) ^ƒ^	5/3 ^ƒ^
	38 (18.5) ^‡^	79 (17.0) ^‡^	4/2 ^‡^
	29 (18.7) ^∆^	32 (17.8) ^∆^	4/1 ^∆^
Clinical history			
Chronic obstructive lung disease —No. (%)	4 (1.6) *	2 (0.9) *	9/5 *
	4 (1.6) ^ƒ^	2 (0.9) ^ƒ^	9/5 ^ƒ^
	4 (2.0) ^‡^	1 (0,5) ^‡^	8/5 ^‡^
	3 (1.9) ^∆^	1 (0.6) ^∆^	5/4 ^∆^
Interstitial lung disease —Quantity (%)	1 (0.4) *	0 (0) *	9/7 *
	1 (0.4) ^ƒ^	0 (0) ^ƒ^	9/7 ^ƒ^
	1 (0.4) ^‡^	0 (0) ^‡^	8/6 ^‡^
	1 (0.4) ^∆^	0 (0) ^∆^	5/6 ^∆^
Pulmonary embolism —No. (%)	1 (0.4) *	1 (0.5) *	10/6 *
	1 (0.4) ^ƒ^	1 (0.5) ^ƒ^	10/6 ^ƒ^
	1 (0.4) ^‡^	1 (0.5) ^‡^	9/5 ^‡^
	1 (0.4) ^∆^	1(0.5) ^∆^	6/5 ^∆^
Deep vein thrombosis —No. (%)	5 (2.0) *	4 (1.8) *	11/6 *
	5 (2.0) ^ƒ^	4 (1.8) ^ƒ^	11/6 ^ƒ^
	3 (1,5) ^‡^	3 (1.5) ^‡^	10/6 ^‡^
	3 (1.9) ^∆^	3 (1.7) ^∆^	6/5 ^∆^
Bronchial asthma —No. (%)	22 (9.0) *	8 (3.6) *	23/8 *
	22 (9.0) ^ƒ^	8 (3.6) ^ƒ^	23/8 ^ƒ^
	20 (9.9) ^‡^	8 (4.0) ^‡^	16/6 ^‡^
	16 (10.5) ^∆^	5 (2.8) ^∆^	9/6 ^∆^
Myocardial Infarction —No. (%)	4 (1.6) *	4 (1.8) *	0/0 *
	4 (1.6) ^ƒ^	4 (1.8) ^ƒ^	0/0 ^ƒ^
	4 (1.9) ^‡^	3 (1.5) ^‡^	0/0 ^‡^
	4 (2.6) ^∆^	3 (1.7) ^∆^	0/0 ^∆^
Transient ischemic attack/stroke —No. (%)	3 (1.2) *	5 (2.3) *	1/0 *
	3 (1.2) ^ƒ^	5 (2.3) ^ƒ^	1/0 ^ƒ^
	3 (1.5) ^‡^	5 (2.5) ^‡^	1/0 ^‡^
	1 (0.7) ^∆^	4 (2.2) ^∆^	0/0 ^∆^
Coronary artery disease —No. (%)	5 (2.0) *	7 (3.2) *	0/0 *
	5 (2.0) ^ƒ^	7 (3.2) ^ƒ^	0/0 ^ƒ^
	5 (2.5) ^‡^	5 (2.5) ^‡^	0/0 ^‡^
	5 (2.5)	5 (3.2)	0/0 ^∆^
Peripheral artery disease —No. (%)	4 (1.6) *	2 (0.9) *	1/0 *
	4 (1.6) ^ƒ^	2 (0.9) ^ƒ^	1/0 ^ƒ^
	3 (1.5) ^‡^	1 (0.5) ^‡^	0/0 ^‡^
	3 (1.9) ^∆^	1 (0.6) ^∆^	0/0 ^∆^

Results are given as absolute value with (percentage)/mean ± standard deviation. (*) initial visit, (*^f^*) 1st year visit, (^‡^) 2nd year visit, (^Δ^) 3rd year visit.

**Table 3:** (Re-)Infection rates.

	SARS-CoV-2 Cohort			Control Cohort		
	1st Year FU n = 245	2nd Year FU n = 206	3rd Year FU n = 155	1st Year FU n = 221	2nd Year FU n = 200	3rd Year FU n = 179
(Re)-Infection SARS-CoV-2	50 (20.6)	92 (44.7)	48 (31.2)	69 (31.2)	121 (60.5)	73 (41.0)
Missing value	2	3	1	0	4	3
Hospitalization 1EP	23 (9.4)	12 (5.8)	17 (11.0)	17 (7.7)	16 (8.0)	6 (3.4)
Loss of Follow-Up	-	36	54	-	19	23

Results are given as absolute value with (percentage).

**Table 4:** Vaccinations against SARS-CoV-2 after initial study inclusion.

	SARS-CoV-2 Cohort		Control Cohort	
	1st Year Visit n = 245	2nd Year Visit n = 206	1st Year Visit n = 221	2nd Year Visit n = 200
1. (Basic immunization)	228 (93.1)	3 (1.4)	188 (85.1)	3 (1.5)
2. (Basic immunization)	195 (79.6)	5 (2.4)	182 (82.4)	3 (1.0)
3. (1. Booster)	40 (16.3)	15 (7.2)	159 (71.9)	1 (0.5)
4. (2. Booster)	1 (0.4)	7 (3.4)	6 (2.7)	30 (14.9)
5. (3. Booster)	-	2 (1.0)	-	5 (2.5)
6. (4. Booster)	-	-	-	2 (1.0)

Results are given as absolute value with (percentage).

**Table 5:** Clinical events leading to hospitalization.

	**SARS-CoV-2 Cohort**		**Control Cohort**	
	initial visit * n = 245		initial visit * n = 221	
	1st year ^ƒ^ n = 245		1st year ^ƒ^ n = 221	
	2nd year ^‡^ n = 206		2nd year ^‡^ n = 200	
	3rd year ^∆^ n = 155		3rd year ^∆^ n = 179	
**Clinical events—Cumulative (%)**	57 (6.7)		45 (5.5)	
**Clinical events—No. (%)**	5 (2.0) *		6 (2.7) *	
	23 (9.4) ^ƒ^		17 (7.7) ^ƒ^	
	12 (5.8) ^‡^		16 (8.0) ^‡^	
	17 (11.0) ^∆^		6 (3.4) ^∆^	
**Classification**	Frequency	Examples of admission diagnosis	Frequency	Examples of admission diagnosis
**Cardiology/Pulmonology/Neurology** **Internal medicine**	3 (1.2) * 8 (3.3) ^ƒ^ 5 (2.4) ^‡^ 5 (3.2) ^∆^	Coronary heart disease, angina pectoris, tachyarrhythmia absoluta, post-COVID-19 pneumonia syndrome, paramedian hypesthesia; vestibular neuritis	2 (0.9) * 7 (3.2) ^ƒ^ 5 (2.5) ^‡^ 2 (1.1) ^∆^	Coronary heart disease, stable angina, heart failure, tachymyopathy, myocarditis, tachyarrhythmia absoluta
**Orthopedics/Surgery**	2 (0.8) * 10 (4.1) ^ƒ^ 5 (2.4) ^‡^ 7 (4.5) ^∆^	Spinal canal stenosis, torn ligaments, contusion, hallux, arthrosis, lumbago, subdural hematoma, clavicle fracture	3 (1.4) * 6 (2.7) ^ƒ^ 5 (2.5) ^‡^ 3 (1.7) ^∆^	Olecranon/femur fracture, lumbago, meniscus lesion, gonarthrosis, supraspinatus defect
**Other (Psychiatry/ENT/Urology/Dermatology/Gynecology)**	0 (0.0) * 5 (2.0) ^ƒ^ 2 (1.0) ^‡^ 5 (3.2) ^∆^	Atopic dermatitis, oropharyngeal carcinoma, urolithiasis, depression, fatigue, endometriosis	1 (0.5) * 4 (1.8) ^ƒ^ 6 (3.0) ^‡^ 1 (0.6) ^∆^	Anaphylaxis, urosepsis, ovarian cyst rupture, prostate, cyst adenoma

Results are given as absolute value with (percentage). (*) initial visit, (*^f^*) 1st year visit, (^‡^) 2nd year visit, (^Δ^) 3rd year visit.

**Table 6:** Short form (SF-36) health questionnaire.

		SARS-CoV-2 Initial Visit n = 245 1st Year n = 245 2nd Year n = 206	Control Initial Visit = 221 1st Year n = 221 2nd Year n = 200	Difference in Means	Missing Values SARS-CoV-2/Control
SF-36 (Short Form 36) Summary Scores					
Physical Component Score (0–100)	Initial Visit	54.2 ± 9.4	57 ± 7.4	3.24 [0.58; 5.91]	9/3
	1st year visit	53.1 ± 10.2	55.0 ± 9.7	1.93 [−1.03; 4.88]	58/39
	2nd year visit	52 ± 10.6	53.9 ± 11.0	1.15 [−2.07; 4.38]	95/61
Mental Component Score (0–100)	Initial Visit	46.2 ± 12.0	51.5 ± 9.7	5.29 [2.01; 8.56]	9/3
	1st year visit	45.1 ± 12.9	49.0 ± 9.7	3.81 [0.17; 7.44]	58/39
	2nd year visit	45.9 ± 12.0	49.6 ± 11.9	3.70 [−0.26; 7.66]	95/61
SF-36 (Short Form 36) Sub Scores					
Physical Functioning Score (0–100)	Initial Visit	87.5 ± 16.4	93.1 ± 11.8	5.63 [0.82; 10.45]	7/1
	1st year visit	86.5 ± 20.0	90.8 ± 17.3	4.39 [−0.64; 9.43]	28/20
	2nd year visit	86.1 ± 18.7	89.0 ± 20.1	2.86 [−2.56; 8.29]	63/43
Role Functioing/ Physical Score (0–100)	Initial Visit	76.5 ± 33.5	91.7 ± 22.8	15.2 [6,54; 23.86]	7/0
	1st year visit	74.4 ± 36.1	84.7 ± 30.5	10.02 [1.06; 18.99]	17/15
	2nd year visit	78.6 ± 33.7	83.0 ± 31.7	5.39 [−4.17; 14.96]	55/31
Role Functioning/Emotional Score (0–100)	Initial Visit	73.8 ± 37.4	86.6 ± 29.0	12.79 [3.24; 23.35]	7/2
	1st year visit	73.5 ± 38.6	81.1 ± 32.3	7.04 [−2.70; 16.78]	15/11
	2nd year visit	76.4 ± 36.6	81.8 ± 33.2	5.58 [−4.88; 16.03]	56/29
Energy/Fatigue Score (0–100)	Initial Visit	56.0 ± 23.3	67.2 ± 21.5	11.17 [4.93; 17.42]	0/0
	1st year visit	52.3 ± 22.4	62.9 ± 23.5	10.68 [4.20; 17.16]	18/15
	2nd year visit	52.7 ± 10.6	61.3 ± 24.0	8.54 [1.60; 15.48]	57/33
Emotional Well-being Score (0–100)	Initial Visit	73.4 ± 20.4	80.6 ± 15.4	7.16 [2.27; 12.05]	0/1
	1st year visit	71.0 ± 18.8	77.0 ± 19.2	6.02 [0.96; 11.08]	20/12
	2nd year visit	71.5 ± 17.6	76.5 ± 19.0	5.08 [−0.34; 10.49]	58/30
Social Functioning Score (0–100)	Initial Visit	83.7 ± 22.7	90.0 ± 18.6	6.25 [0.44; 12.06]	5/1
	1st year visit	81.8 ± 22.7	87.4 ± 21.3	5.72 [−0.19; 11.64]	12/10
	2nd year visit	85.5 ± 20.0	87.7 ± 20.9	2.17 [−4.12; 8.46]	49/25
Pain Score (0–100)	Initial Visit	85.0 ± 22.7	89.7 ± 18.3	4.72 [−1.60; 11.03]	6/1
	1st year visit	78.9 ± 25.1	82.7 ± 23.9	3.82 [−2.60; 10.25]	14/8
	2nd year visit	82.0 ± 22.4	81.2 ± 24.8	−0.71 [−7.57; 6.13]	50/26
General Health Score (0–100)	Initial Visit	72.8 ± 20.4	79.2 ± 17.8	6.39 [0.75; 12.03]	0/1
	1st year visit	69.2 ± 21.0	73.9 ± 21.0	4.55 [−1.25; 10.36]	13/13
	2nd year visit	68.1 ± 22.5	72.4 ± 21.4	4.36 [−1.91; 10.63]	59/31

Data are given as mean ± standard deviation; in square brackets, confidence intervals are given. Significant differences in means/odds ratio are written in bold type.

**Table 7:** Results of PCFS-Scale.

		SARS-CoV-2 Initial Visit n = 245 1st Year n = 245 2nd Year n = 206	Control Initial Visit = 221 1st Year n = 221 2nd Year n = 200	Missing Values SARS-CoV-2/Control	Odds Ratio 95% CI
PCFS (Post-COVID-Functional Scale) derived from EQ-5D-5L, SF-36					
**PCFS < 2** **—No. (%)**	Initial Visit	167 (68.2)	188 (85.1)	-	Initial Visit OR 2.66 [1.68; 4.20] *p* < 0.0001 1st year Follow-up OR 1.70 [1.12; 2.57] *p* = 0.012 2nd year Follow-up OR 1.33 [0.85; 2.08] *p* = 0.203
	1st year Follow-up	159 (66.5)	169 (77.2)	6/2	
	2nd year Follow-up	182 (75.5)	177 (80.5)	4/1	
**PCFS > 2** **—No. (%)**	Initial Visit	78 (31.8)	33 (14.9)	-	
	1st year Follow-up	80 (33.5)	50 (22.8)	6/2	
	2nd year Follow-up	59 (24.5)	43 (19.5)	4/1	
**PCFS (Post-COVID-Functional Scale) original**					
**PCFS < 2** **—No. (%)**	Initial Visit	-	-	-	1st year Follow-up OR 1.52 [1.03; 2.26] *p* = 0.035 2nd year Follow-up OR 1.35 [0.89; 2.03] *p* = 0.16
	1st year Follow-up	155 (63.3)	160 (72.4)	-	
	2nd year Follow-up	172 (70.2)	168 (76.0)	-	
**PCFS > 2** **—No. (%)**	Initial Visit	-	-	-	
	1st year Follow-up	90 (36.7)	61 (27.6)	-	
	2nd year Follow-up	73 (29.8)	53 (24.0)90	-	

Data are given as mean ± standard deviation, and 95% confidence intervals are in square brackets.

## Data Availability

The data used to support this study are available in https://redcap.glkn.de/surveys/?__report=PE93J8XED8HR7MW7 (accessed on 19 January 2025).

## Supplementary Materials

The following supporting information can be downloaded at: https://www.mdpi.com/article/10.3390/jcm14051439/s1.

## References

[1] Kumar R., Aktay-Cetin Ö., Craddock V., Morales-Cano D., Kosanovic D., Cogolludo A., Perez-Vizcaino F., Avdeev S., Kumar A., Ram A.K. (2023). Potential long-term effects of SARS-CoV-2 infection on the pulmonary vasculature: Multilayered cross-talks in the setting of coinfections and comorbidities. PLoS Pathog..

[2] Giovanetti M., Benedetti F., Campisi G., Ciccozzi A., Fabris S., Ceccarelli G., Tambone V., Caruso A., Angeletti S., Zella D. (2021). Evolution patterns of SARS-CoV-2: Snapshot on its genome variants. Biochem. Biophys. Res. Commun..

[3] Aleem A., Ab A.S., Slenker A.K. (2025). Variants of SARS-CoV-2 and Novel Therapeutics Against Coronavirus (COVID-19). StatPearls [Internet].

[4] Hernández-Aceituno A., García-Hernández A., Larumbe-Zabala E. (2023). COVID-19 long-term sequelae: Omicron versus Alpha and Delta variants. Infect. Dis. Now..

[5] Rabaan A.A., Smajlović S., Tombuloglu H., Cordic S., Hajdarevic A., Kudic N. (2022). SARS-CoV-2 infection and multi-organ system damage: A review. Bosn. J. Basic Med. Sci..

[6] Koczulla A.R., Ankermann T., Behrends U., Berlit P., Berner R., Böing S., Brinkmann F., Frank U., Franke C., Glöckl R. (2022). S1-Leitlinie Long-/Post-COVID. Pneumologie.

[7] Martinez-Rojas M.A., Vega-Vega O., Bobadilla N.A. (2020). Is the kidney a target of SARS-CoV-2?. Am. J. Physiol. Ren. Physiol..

[8] Gabarre P., Dumas G., Dupont T., Darmon M., Azoulay E., Zafrani L. (2020). Acute kidney injury in critically ill patients with COVID-19. Intensive Care Med..

[9] Chen R., Wang K., Yu J., Howard D., French L., Chen Z., Wen C., Xu Z. (2021). The Spatial and Cell-Type Distribution of SARS-CoV-2 Receptor ACE2 in the Human and Mouse Brains. Front. Neurol..

[10] Huang C., Wang Y., Li X., Ren L., Zhao J., Hu Y., Zhang L., Fan G., Xu J., Gu X. (2020). Clinical features of patients infected with 2019 novel coronavirus in Wuhan, China. Lancet.

[11] Gupta A., Madhavan M.V., Sehgal K., Nair N., Mahajan S., Sehrawat T.S., Bikdeli B., Ahluwalia N., Ausiello J.C., Wan E.Y. (2020). Extrapulmonary manifestations of COVID-19. Nat. Med..

[12] Elmunzer B.J., Spitzer R.L., Foster L.D., Merchant A.A., Howard E.F., Patel V.A., West M.K., Qayed E., Nustas R., Zakaria A. (2021). Digestive Manifestations in Patients Hospitalized With Coronavirus Disease 2019. Clin. Gastroenterol. Hepatol..

[13] Xu L., Liu J., Lu M., Yang D., Zheng X. (2020). Liver injury during highly pathogenic human coronavirus infections. Liver Int..

[14] Mikolajczyk R., Diexer S., Klee B., Pfrommer L., Purschke O., Fricke J., Ahnert P., Gabrysch S., Gottschick C., Bohn B. (2024). Likelihood of Post-COVID Condition in people with hybrid immunity; data from the German National Cohort (NAKO). J. Infect..

[15] O’Mahoney L.L., Routen A., Gillies C., Ekezie W., Welford A., Zhang A., Karamchandani U., Simms-Williams N., Cassambai S., Ardavani A. (2022). The prevalence and long-term health effects of Long Covid among hospitalised and non-hospitalised populations: A systematic review and meta-analysis. EClinicalMedicine.

[16] Haberland E., Haberland J., Richter S., Schmid M., Hromek J., Zimmermann H., Geng S., Winterer H., Schneider S., Kollum M. (2022). Seven Months after Mild COVID-19: A Single-Centre Controlled Follow-Up Study in the District of Constance (FSC19-KN). Int. J. Clin. Pract..

[17] Harris P.A., Taylor R., Thielke R., Payne J., Gonzalez N., Conde J.G. (2009). Research electronic data capture (REDCap)—A metadata-driven methodology and workflow process for providing translational research informatics support. J. Biomed. Inform..

[18] Harris P.A., Taylor R., Minor B.L., Elliott V., Fernandez M., O’Neal L., McLeod L., Delacqua G., Delacqua F., Kirby J. (2019). The REDCap Consortium: Building an International Community of Software Platform Partners. J. Biomed. Inform..

[19] Halpin D.M.G., Singh D., Hadfield R.M. (2020). Inhaled corticosteroids and COVID-19: A systematic review and clinical perspective. Eur. Respir. J..

[20] Adir Y., Saliba W., Beurnier A., Humbert M. (2021). Asthma and COVID-19: An update. Eur. Respir. Rev..

[21] Griesel M., Wagner C., Mikolajewska A., Stegemann M., Fichtner F., Metzendorf M.I., Nair A.A., Daniel J., Fischer A.L., Skoetz N. (2022). Inhaled corticosteroids for the treatment of COVID-19. Cochrane Database Syst. Rev..

[22] Hsu C.-W., Lee M.-C., Hua Y.-M., Lai C.-C., Tang H.-J., Chao C.-M. (2023). Inhaled corticosteroid for patients with COVID-19: A systematic review and meta-analysis of randomized controlled trials. J. Microbiol. Immunol. Infect..

[23] Shin J.I., Kim S.E., Lee M.H., Kim M.S., Lee S.W., Park S., Shin Y.H., Yang J.W., Song J.M., Moon S.Y. (2022). COVID-19 susceptibility and clinical outcomes in autoimmune inflammatory rheumatic diseases (AIRDs): A systematic review and meta-analysis. Eur. Rev. Med. Pharmacol. Sci..

[24] You Y., Yang X., Hung D., Yang Q., Wu T., Deng M. (2024). Asymptomatic COVID-19 infection: Diagnosis, transmission, population characteristics. BMJ Support. Palliat. Care.

[25] Buitrago-Garcia D., Ipekci A.M., Heron L., Imeri H., Araujo-Chaveron L., Arevalo-Rodriguez I., Ciapponi A., Cevik M., Hauser A., Alam M.I. (2022). Occurrence and transmission potential of asymptomatic and presymptomatic SARS-CoV-2 infections: Update of a living systematic review and meta-analysis. PLoS Med..

[26] McLaughlin J.M., Khan F., Pugh S., Swerdlow D.L., Jodar L. (2022). County-level vaccination coverage and rates of COVID-19 cases and deaths in the United States: An ecological analysis. Lancet Reg. Health Am..

[27] Wrenn J.O., Pakala S.B., Vestal G., Shilts M.H., Brown H.M., Bowen S.M., Strickland B.A., Williams T., Mallal S.A., Jones I.D. (2022). COVID-19 severity from Omicron and Delta SARS-CoV-2 variants. Influ. Other Respir. Viruses.

[28] Shen X., Wang P., Shen J., Jiang Y., Wu L., Nie X., Liu J., Chen W. (2023). Neurological Manifestations of hospitalized patients with mild to moderate infection with SARS-CoV-2 Omicron variant in Shanghai, China. J. Infect. Public Health.

[29] Kashif A., Chaudhry M., Fayyaz T., Abdullah M., Malik A., Anwer J.M., Inam S.H., Fatima T., Iqbal N., Shoaib K. (2021). Follow-up of COVID-19 recovered patients with mild disease. Sci. Rep..

[30] Wahlgren C., Forsberg G., Divanoglou A., Balkhed Å.Ö., Niward K., Berg S., Levi R. (2023). Two-year follow-up of patients with post-COVID-19 condition in Sweden: A prospective cohort study. Lancet Reg. Health Eur..

[31] Crook H., Raza S., Nowell J., Young M., Edison P. (2021). Long covid—Mechanisms, risk factors, and management. BMJ.

[32] Wynberg E., Han A.X., Boyd A., van Willigen H.D., Verveen A., Lebbink R., van der Straten K., Kootstra N., van Gils M.J., Russell C. (2022). The effect of SARS-CoV-2 vaccination on post-acute sequelae of COVID-19 (PASC): A prospective cohort study. Vaccine.

[33] Park J.M., Woo W., Lee S.C., Park S., Yon D.K., Lee S.W., Smith L., Koyanagi A., Shin J.I., Kim Y.W. (2023). Prevalence and Mortality Risk of Neurological Disorders during the COVID-19 Pandemic: An Umbrella Review of the Current Evidence. Neuroepidemiology.

[34] Jassat W., Karim S.S.A., Mudara C., Welch R., Ozougwu L., Groome M.J., Govender N., von Gottberg A., Wolter N., Wolmarans M. (2022). Clinical severity of COVID-19 in patients admitted to hospital during the omicron wave in South Africa: A retrospective observational study. Lancet Glob. Health.

[35] Antonelli M., Pujol J.C., Spector T.D., Ourselin S., Steves C.J. (2022). Risk of long COVID associated with delta versus omicron variants of SARS-CoV-2. Lancet.

